# Thermally Self-Healing Graphene-Nanoplate/Polyurethane Nanocomposites via Diels–Alder Reaction through a One-Shot Process

**DOI:** 10.3390/nano9030434

**Published:** 2019-03-14

**Authors:** Cho-Rong Oh, Sang-Hyub Lee, Jun-Hong Park, Dai-Soo Lee

**Affiliations:** 1 Division of Semiconductor and Chemical Engineering, Chonbuk National University, Baekjedaero 567, Jeonju 54896, Korea; ohcho38@naver.com (C.-R.O.); shlee87@jbnu.ac.kr (S.-H.L.); 2 R & D Center, Lotte Advanced Materials, Sandan-ro 334-27, Yeosu 59616, Korea

**Keywords:** graphene-nanoplate, polyurethane, nanocomposite, Diels–Alder reaction, self-healing

## Abstract

Thermally self-healing graphene-nanoplate/polyurethane (GNP/PU) nanocomposites were prepared via a bulk in-situ Diels–Alder (DA) reaction. Graphene-nanoplate (GNP) was used as a reinforcement and crosslinking platform by a DA reaction with a furfuryl-based chain extender of polyurethane (PU). Results showed that a DA reaction occurred in GNP during the PU forming cure process. This procedure is simple and solvent free because of the absence of any independent surface modification process. Through the calculation of the interfacial tensions, the conditions of the bulk in-situ DA reaction were determined to ensure that GNP and the furfuryl group can react with each other at the interface during the curing process without a solvent. The prepared composites were characterized in terms of thermal, mechanical, and thermally self-healing properties via the DA reaction. In the PU capable of a DA reaction (DPU), characteristic peaks of DA and retro DA reactions were observed in the Fourier transform infrared (FT-IR) spectroscopy and endothermic peaks of retro DA reactions appeared in differential scanning calorimetry (DSC) thermograms. The DPU showed significantly enhanced physical properties and chemical resistance. The thermally self-healing capability was confirmed at 110 °C via the retro DA reactions. It is inferred that thermally self-healable crosslinked GNP/PU nanocomposites via DA reactions could be prepared in a simple bulk process through the molecular design of a chain extender for the in-situ reaction at the interface.

## 1. Introduction

Polymers in crosslinked structures offer considerable advantages such as improvements to heat and chemical resistance and mechanical strength. However, reprocessing or recycling crosslinked polymers is difficult because of the limited diffusivity. Recently, much research has reported network polymers that are self-healable by stimuli and remendable based on the dynamic covalent bonds from Diels–Alder (DA) reaction adducts, disulfides, and imines under mild conditions [1,2,3].

The DA reaction is the most conventional dynamic covalent bond and can mildly control the reversible condition. It is a reaction between a diene and dienophile and is reversible depending on temperature. Furfuryl compounds are generally used as a diene, and maleimide is used as a dienophile because it can be modified in various ways. Furthermore, homopolymerization of the maleimide can continue when a system is exposed to temperatures above 150 °C. Hence, the main process should be conducted at mild temperatures [4,5,6]. Polyurethane (PU) is one of the most versatile polymers, and it can be used for various applications. The versatilities of PUs are based on the variety of constituents such as types of polyols, isocyanates, and chain extenders or crosslinkers for PUs. Thus, PU is one of the most favorable polymers for which the self-healing functional compounds can be introduced.

Graphene consists of a single atomic layer of sp^2^ carbon. It can be fabricated via various exfoliation methods from graphite [7,8]. Graphene has exceptional electrical, thermal, and mechanical properties, and it can substantially enhance polymer properties at a much lower content than conventional fillers [9,10]. Furthermore, graphene can be used as a reactant in a DA reaction [11,12,13,14,15,16,17]. Haddon et al. have recently reported that single-layer graphene can be used in DA and retro DA reactions as a dienophile or diene in combination with another reactant at various temperatures [11]. Several researchers succeeded in the exfoliation of graphite via a DA reaction of tetracyanoethylene and graphite and synthesized graphene oxide via a DA reaction between graphene and maleic anhydride [12,13]. The DA reaction between the carbon-based material and small molecules, as well as the polymer, was also investigated [14,15]. Bai et al. reported a crosslinked poly(styrene-b- butadiene-b-styrene) (SBS) via a DA reaction between C-60 and furan-modified SBS and its thermally self-healing properties [16]. Hager et al. reported that poly(lauryl methacrylate) with anthracene moieties in the side chain was converted with C-60 and phenyl-C61-butyric acid methyl ester, thereby producing a self-healing polymeric material [17].

The abovementioned studies required the use of an additional process for the DA reaction with graphene and needed a solvent for the dispersion of graphene. However, the solvent-casting method results in volatile organic compound problems during the drying process. In our previous study, we highlighted how preparing a masterbatch, in which graphene is dispersed in polyol, can solve the problem of the poor dispersion of graphene in PU [18,19].

To reduce the process for surface modification of graphene, an in-situ DA reaction should occur during the curing process of polymers to produce a crosslinked PU via a DA reaction. Interfacial tensions are used to determine the degree of interfacial wetting and adhesion. Graphene and the reactant must be in contact for the DA reaction during the curing process. Hence, the interfacial tension between graphene and the reactant of DA reaction should be smaller than the sum of the interfacial tension between the matrix and the reactant, and that between the graphene and the matrix. The interfacial tension can be calculated on the basis of the surface tension of each material. The commonly used interfacial tension relationships are those of Wu and Owen–Wendt given in Equations (1) and (2), respectively. These formulas consider the dispersion and polar components of the surface tensions in accordance with the modified form of Fowke’s equations [20,21,22] as follows;

(1) $$\gamma_{S-l}=\gamma_{S}+\gamma_{l}-\frac{4\gamma_{S}^{d}\gamma_{l}^{d}}{\gamma_{S}^{d}+\gamma_{l}^{d}}-\frac{4\gamma_{S}^{p}\gamma_{l}^{p}}{\gamma_{S}^{p}+\gamma_{l}^{p}}$$

(2) $$\gamma_{S-l}=\gamma_{s}+\gamma_{l}-2{\left(\gamma_{s}^{d}\gamma_{l}^{d}\right)}^{\frac{1}{2}}-2{\left(\gamma_{s}^{p}\gamma_{l}^{p}\right)}^{\frac{1}{2}}$$

(3) $$\gamma_{i}=\gamma_{i}^{d}+\gamma_{i}^{p}$$

where $\gamma_{i}$ is the surface tension of material (*i*); $\gamma_{S-l}$ is the interfacial tension between solid (*s*) and liquid (*l*); and $\gamma_{i}^{d}$ and $\gamma_{i}^{p}$ the dispersive and polar components of $\gamma_{i}$, respectively.

In this study, a concentrate of graphene-nanoplate (GNP) was obtained by dispersing GNP in a polyol for PU and the nanocomposites of PU and GNP were prepared via an in-situ DA reaction without any surface treatment of GNP. Importantly, a masterbatch was prepared by concentrating GNP in the polyol to ensure its good dispersibility. The interfacial tension between the materials was calculated so as to ensure that GNP was wet with the furfuryl compound and synthesized to enable the DA reaction with GNP without solvent. The mechanical properties and chemical resistance of the obtained composites were enhanced through the crosslinking effect by DA reaction. In addition, thermally self-healing properties were noticed in the nanocomposites via retro DA reactions.

## 2. Experimental

### 2.1. Materials

Poly(tetramethylene ether glycol) (PTMEG; Mn = 1000 g/mol), 4,4‘-methylenebis(phenyl isocyanate) (MDI), 1,4-butanediol (BD), and furfuryamine (FA) were purchased from Sigma-Aldrich (Young-in, Korea). Glycerol carbonate (GC) was supplied by Huntsman (Seoul, Korea). N-methylpyrrolidone (NMP) and dimethyl acetamide (DMAc) were purchased from SK Chemical (Seongnam, Korea). GNP was purchased from Carbon Nano-material Technology (Pohang, Korea). PTMEG was dried in a vacuum oven at 60 °C for 1 day before use. Meanwhile, the BD was dried with molecular sieves for 1 day.

### 2.2. Preparation of GNP Masterbatch

The masterbatch, which was concentrated GNP in PTMEG, was prepared to facilitate the dispersion of GNP. A total of 1 g of GNP and 1 L of NMP were mixed into 2 L beaker. The mixture was sonicated for 1 day. After the sonication, the mixture was centrifugated at 3000 rpm for 45 min [23]. The supernatant, which was obtained after centrifugation, and 100 g of PTMEG were mixed in a round-bottom flask and magnetically stirred for 1 h. The concentrated GNP in PTMEG was formed after the removal of NMP at 200 °C through evaporation. The supernatants were mixed with the concentrated GNP in PTMEG repeatedly and the final GNP content of the masterbatch was 5 wt%. The obtained masterbatch was dried in a vacuum oven at 60 °C for 3 days before use.

### 2.3. Preparation of GNP/PU Nanocomposites

The mole ratio of GC to FA of 1:1 was fed into a reactor to obtain the furfuryl derivative (FD). The mixture was reacted at 80 °C for 3 h in N_2_ atmosphere. The obtained FD was dried in a vacuum oven at 60 °C for 1 day before use and characterized by titration, Fourier transform infrared (FT-IR) spectrum, and ^1^H nuclear magnetic resonance (^1^H-NMR) spectrum. The GNP content of GNP/PU nanocomposites varied between 0 to 2 wt%, in which the masterbatch was diluted by the addition of neat polyol. A total of 1 mol of PTMEG, which contained a masterbatch, and 2 mol of MDI were reacted in the reactor to prepare the prepolymer at 60 °C until the theoretical isocyanate content was reached. The stoichiometric amount of chain extender, such as BD or FD, was added into the prepolymer and mixed for 1 min. Following this, the mixture was cast into a glass mold and cured at 70 °C for 3 days. The sample was called x-CPU or x-DPU, where “x” indicates the weight percentage of GNP in the PU nanocomposites. CPU is control PU chain extended with BD. Sample codes and compositions of GNP/PU nanocomposites are given in Table S1.

### 2.4. Characterizations

The Raman spectra were recorded with a Micro-Raman spectrometer (Nanofinder 30), using a 633 nm wavelength laser. The high-resolution transmission electron microscopy (HR-TEM) was performed with JEM 2010 system (JEOL, Akishima, Japan). A thermogravimetric analysis (TGA) of FD was performed using Q600 (TA INST., New Castle, DE, USA) at a heating rate of 20 °C/min under N_2_ atmosphere. The FT-IR spectra were recorded between 4000 and 400 cm^−1^ at a resolution of 4 cm^−1^ in 50 scans using FT-IR-300E (JASCO, Easton, MD, Japan). The samples were prepared by dropping the solution onto a pure KBr pellet. ^1^H-NMR spectra were obtained using the ECA600 Fourier transform high-resolution nuclear magnetic resonance spectrometer (JEOL, Akishima, Japan). Tetramethylsilane was used as an internal standard in DMSO-*d6*. Contact angle measurements were performed employing a device, Phoenix 150 (SEO, Suwon, Korea). The static contact angles of liquids were measured by depositing a liquid drop of 3 to 5 μL on the surface, and the values were recorded at the normal tangent of the drop at the intersection between the liquid and solid surface. Pictures were captured within 30 s of the drop deposition and contact angle values were reported to be the average of at least five tests at different spots of the surface. Differential scanning calorimetry (DSC) measurements were performed using Q20 (TA INST., USA). DSC thermograms were recorded in all synthesized polymers, which were sealed in aluminum pans in a dry nitrogen atmosphere, with an empty aluminum pan used as a reference. The scanning was performed at a rate of 10 °C/min. The second heating cycle was recorded after an isothermal process at 60 °C for 1 h. A field emission scanning electron microscope (FE-SEM, JEOL JSM-6400) in combination with an energy-dispersive X-ray analysis (EDX) (SUPRA 40VP, Carl Zeiss, Oberkochen, Germany) was used to observe the elements on the GNP surface. Dumbbell specimens with a width of 5 mm and length of 30 mm were cut from the cast films. Tensile test of the film was performed using the LLOYD LR5K PLUS instrument at a crosshead speed of 500 mm/min at room temperature (RT). A scanning electron microscope (SEM, AIS2100C, Seron Tech Inc., Uiwang, Korea) was utilized for the scratch-healing test in which the cuts were marked at the middle of the films using a razor blade. The gel fractions and swelling ratio of the sample were determined by soaking the samples in DMAc for 48 h at RT. The gel fraction and swelling ratio were calculated using the following equations;

(4) $$Swelling\;ratio=W_{s}/W_{i}$$

(5) $$Gel\;fraction=W_{d}/W_{i}\times 100$$

where $W_{i}$ is the weight of sample before swelling, $W_{s}$ is the weight of insoluble polymer in DMAc and $W_{d}$ is the weight of dried swollen sample at 110 °C. Dynamic mechanical analyses (DMA) were performed using Q800 (TA INST., US). The scanning was performed at a rate of 5 °C /min under a nitrogen atmosphere.

## 3. Results and Discussion

### 3.1. Characterization of GNP and FD

**Figure 1:**
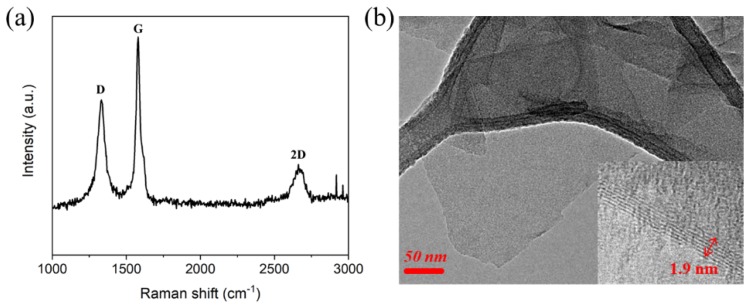
Raman spectra (**a**) and high-resolution transmission electron microscopy (HR-TEM) image (**b**) of a graphene-nanoplate GNP.

The properties of GNP, such as the number of layers and surface defects are important factors in determining the quality of the fabricated graphene. Raman spectroscopy is a very useful tool to analyze carbon-based materials. The Raman spectra of the GNP is shown in Figure 1a. The three main peaks were observed, which were the D band at ~1350 cm^−1^, the G band at ~1580 cm^−1^ and the 2D band at ~2700 cm^−1^. The D band indicates the disorder of the graphene structure attributable to the edge planes and defect in graphene sheets [24]. The intensity ratio of the D band to the G band is related to the concentration of the defect free region in the graphene structure because the G band is due to the whole movement of the sp^2^ carbon atom in the basal plane [25]. The $I_{D}/I_{G}$ for the GNP was calculated to be 0.96. This value is similar to $I_{D}/I_{G}$ for the reduced graphene oxide sheets obtained by hydrazine [26]. The 2D band is related to two D phonon assisted double resonance. Therefore, the intensity ratio of the 2D band to the G band was also used to evaluate the quality and thickness of GNP. Green and Hersam reported that as the thickness of the GNP increases, $I_{2D}/I_{G}$ decreases from a high of 2.1 ± 0.2 for single-layer GNP to 0.8 ± 0.1 for quadruple-layer GNP [27]. The $I_{2D}/I_{G}$ for the GNP was calculated to be 0.91, which corresponded to approximately four layers. The number of layers in the GNP was also confirmed approximately five layers and the area of GNP was several hundred nanometers in Figure 1b.

**Figure 2:**
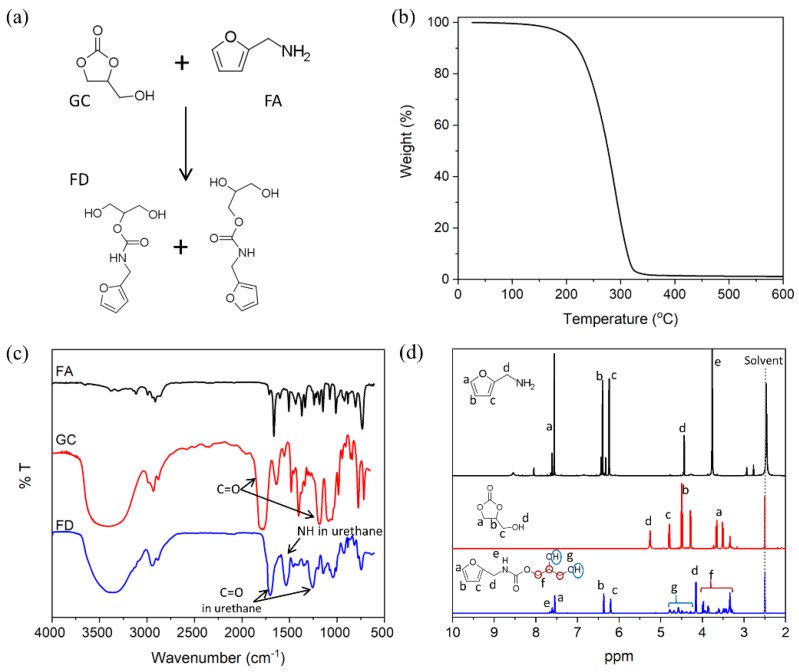
Synthesis and characterization furfuryl derivative (FD): (**a**) Synthesis of FD from glycerol carbonate (GC) and furfuryamine (FA); (**b**) thermogravimetric analysis (TGA) thermogram of the FD; (**c**) Fourier transform infrared (FT-IR) spectra of FA, GC, and FD; (**d**) ^1^H nuclear magnetic resonance (^1^H-NMR) spectra of the FA, GC, and FD.

Figure 2 shows the synthesis and characteristics of FD for DA reactions with GNP. The GC was reacted with a stoichiometric amount of FA at 80 °C to synthesize the FD, which was used as a chain extender with the furfuryl group for the DA reaction. The ring opening reaction of the GC may occur by reacting with a primary amine in FA, thereby forming two hydroxyl groups as shown in Figure 2a. The obtained FD was a transparent red–brown and viscous liquid. The hydroxyl value (ASTM D-4274 D) of the FD was 526.5 mg KOH/g and the yield was 99.04%. In the TGA thermogram given in Figure 2b, the thermal degradation temperature was 200 °C with 5% weight loss. Meanwhile, the thermal stability of FD, which was the chain extender in this study, was satisfactory during the synthesis of PU capable of a DA reaction (DPU). Figure 2c,d show the structural analysis of FD. In the FT-IR spectra shown in Figure 2c, the peak of C=O was observed at 1789 cm^−1^, and that of C–O at 1183 cm^−1^ in GC. After the ring opening reaction, the peak of the carbonate disappeared. Meanwhile, the peaks of C=O (1702 cm^−1^) and C–O (1256 cm^−1^), due to the urethane formed, were observed in FD. Furthermore, the peak of N–H (1535 cm^−1^) was evident. Thus, it was assumed the FD was formed by the reaction between the carbonate of GC and primary amine of FA. FD was also analyzed using the ^1^H-NMR spectrum. A chemical shift of the furfuryl group in FD at δ = 7.55, δ = 6.39, and δ = 6.24 ppm was confirmed at a similar region of the group in FA at δ = 7.54, δ = 6.36, and δ = 6.20 ppm. Thus, the furfuryl group in the FD was fully conserved during the synthesis process.

### 3.2. Diels–Alder Reaction in the Preparation of GNP/PU Nanocomposites

Before confirming the DA reaction in GNP/PU nanocomposites, the TGA thermogram and the Raman spectrum were analyzed to confirm the DA reaction of the GNP and FD. GNP and FD were reacted at 70 °C for a day, washed three times with dimethylformamide and dried and 70 °C for 3 days. The obtained sample was called modified GNP, and Raman spectrum and TGA thermogram of GNP and modified GNP were measured. In the TGA data of Figure S1a, the weight loss of GNP, and that of modified GNP at 600 °C, were 0.6 wt% and 3.5 wt%, respectively. The temperature range of the weight loss of modified GNP was similar to the decomposition temperature range of FD in Figure 2b. This means that modified GNP was produced by the DA reaction between GNP and FD. D band of modified GNP was larger than that of GNP, as shown in the Raman data in Figure S1b. This can be interpreted as a defect in the basal plane of modified GNP due to the DA reaction of GNP and FD.

**Figure 3:**
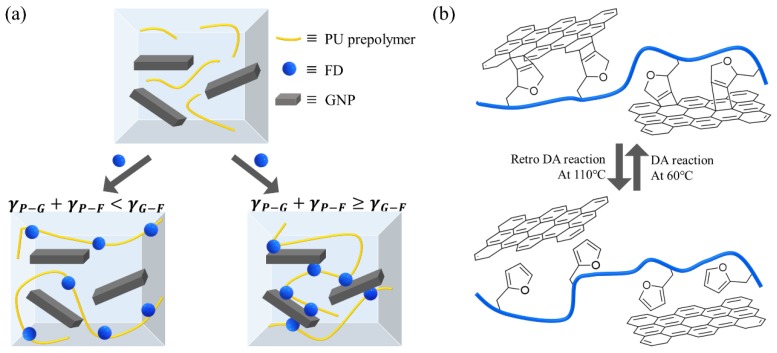
Schematic of two different dispersions in systems (**a**) and DA and retro DA reactions between the GNP and FD in GNP/PU composites (**b**).

DA reactions are prone to occur during the polymerization of PU, when the GNP and FD are in close proximity in the mixture. Figure 3a shows the location of FD with different interfacial tensions in a mixture state. GNP and FD can be close to each other and remain stable when the relationship of the interfacial tension is as follows;

(6) $$\gamma_{GF}<\gamma_{PF}+\gamma_{PG}$$

where $\gamma_{GF}$, $\gamma_{PF}$, and $\gamma_{PG}$ are the interfacial tension between two materials, GNP and FD, prepolymer and FD, and prepolymer and FD respectively. When the above relationship is reversed, the DA reaction is difficult because the PU prepolymer is located around the GNP, and FD is dispersed in the PU prepolymer. $\gamma_{i}$, $\gamma_{i}^{d}$, and $\gamma_{i}^{p}$ are required in order to calculate the interfacial tensions among the reactants. The surface tension values and their components can be calculated from the relationship between the Young’s and Wu’s equation. If the contact angles of at least two liquids with known dispersion and polar component of interfacial tensions are measured on a solid surface, then the $\gamma_{s}^{d}$ and $\gamma_{s}^{p}$, as well as the surface tension of a solid, can be calculated. Using these parameters, the values of the interfacial tension between the two materials can be calculated using Wu’s equation or Owen–Wendt’s equation. The as-obtained surface and interfacial tension values are listed in Table 1, Tables S2 and S3, respectively. It was confirmed that the result of the calculation of the interfacial tension satisfied Equation (6). Hence, the DA reaction may proceed in the polyurethane forming cure process when the PU prepolymer, FD, and GNP are mixed in one shot because GNP can be wet preferentially with FD.

**Figure 4:**
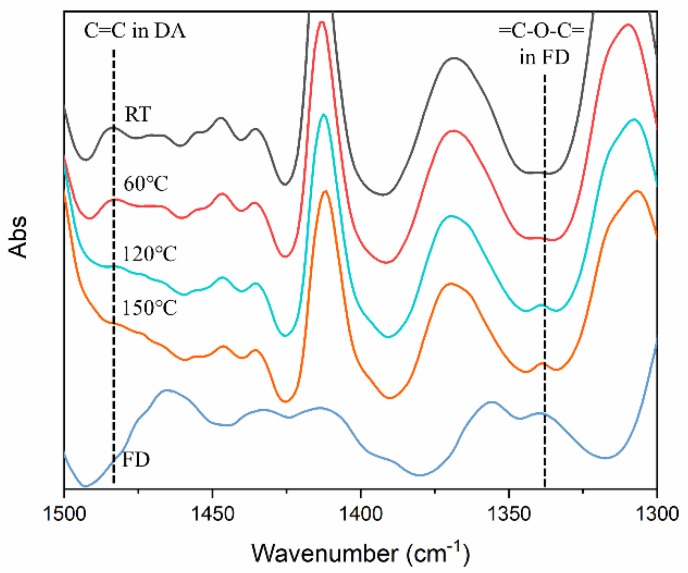
FT-IR spectra of 0.1_DPU at different temperatures and FD at room temperature (RT).

In the DA linkage, one double bond and dialkyl ether are present as shown in Figure 3b. However, the retro DA reactions are expected to generate the conjugated C=C bond and divinyl alkyl ether in FD. Figure 4 shows the FT-IR spectrum of 0.1_DPU at various temperatures. C=C in the DA linkage (1483 cm^−1^) was observed in the spectrum measured at RT and 60 °C. The peak disappeared when the temperature was increased to 120 °C and 150 °C. The peak, due to =C–O–C= in FD (1339 cm^−1^), which was not observed at RT and 60 °C, appeared at 120 °C and 150 °C. The change in peak intensity indicated that the structure of the DA reaction adduct was maintained at RT and 60 °C, and the reverse reaction occurred at 120 °C and 150 °C. Therefore, it was confirmed the DA reaction proceeded well during the polyurethane forming cure process.

**Figure 5:**
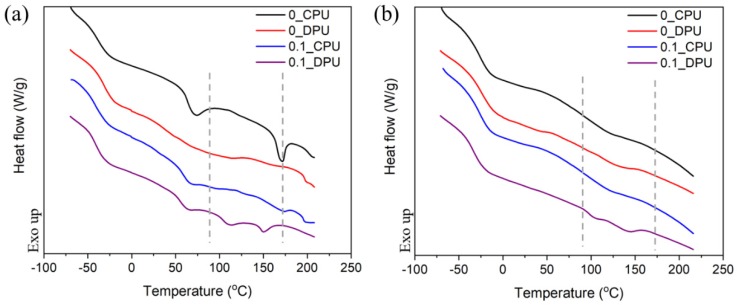
Differential scanning calorimetry (DSC) thermograms of the CPU and DPU with different GNP contents: (**a**) First scan; (**b**) second scan after the isothermal process at 60 ℃ for 1 h.

Figure 5 shows the DSC thermograms of CPU and DPU chain extended with BD and FD, respectively. Enthalpy changes near −50 °C were observed due to the glass transitions of soft segment domains of PUs. Hard segment domains of PUs showed glass-rubbery transitions and crystalline melting at around 50 °C and 170 °C, respectively. It is noteworthy that CPU showed an endotherm of hard segment melting, while DPU did not. It is postulated that CPU underwent microphase separation of hard segments and soft segments extensively, compared with DPU. After the first scan, an isothermal process was performed for 1 h at 60 °C, following which, the second scan was conducted. The most noticeable two endothermic peaks in the primary and secondary scans were at around 100 °C to 150 °C in the DPU systems with both GNP and FD. These endothermic peaks in this range of temperature were attributable to the retro DA reactions discussed based on FT-IR spectra shown in Figure 4.

**Figure 6:**
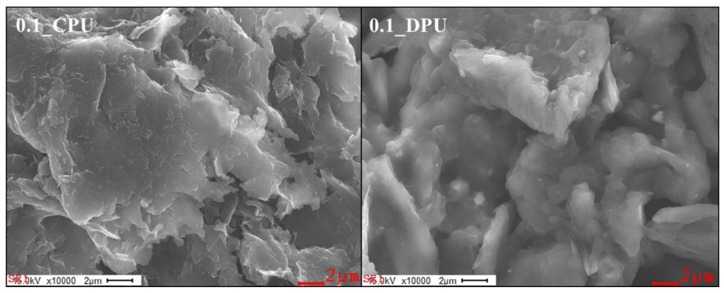
Field emission scanning electron microscope (FE-SEM) images of the GNP collected in 0.1_CPU and 0.1_DPU.

Figure 6 shows the FE-SEM image of the GNPs collected from the nanocomposites, 0.1_DPU and 0.1_CPU. The samples were obtained by filtering and washing after the dissolution of the nanocomposites in DMAc and then dried at 70 °C for 3 days. The GNP surface of 0.1_DPU appeared to be coated with PU polymer, whereas the GNP surface of 0.1_CPU was relatively clean. Table 2 shows the results of the elementary analyses by EDX for GNPs collected. 0.1_DPU showed higher contents of nitrogen and oxygen than 0.1_CPU because of the presence of a PU chains in the GNP surface cross-linked via the DA reactions.

**Figure 7:**
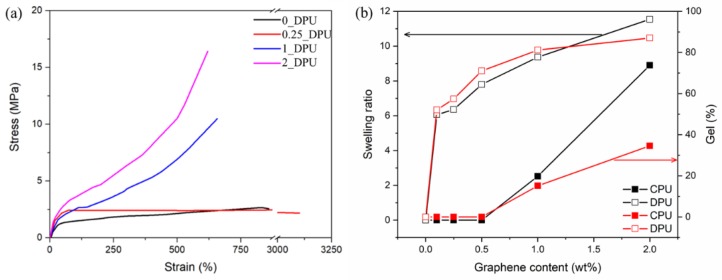
Stress–strain curve of the DPU with various GNP contents (**a**), swelling ratios (open symbols) and gel fractions (filled symbols) of the CPU and DPU with various GNP contents (**b**).

Figure 7a shows the stress–strain curve of DPUs of the different GNP content. As the GNP content increased, the Young’s modulus and tensile strength of the composites increased because of the crosslinking by the DA reaction, as well as the reinforcement effects of GNP. Tensile properties of CPUs of different GNP are shown in Figure S2. It was observed that the mechanical properties of CPUs were superior to those of DPUs due to extensive microphase separation of hard segments and soft segments in PU chains. The crosslinking effect of the DA reaction can be confirmed through a swelling test. A gel was observed at 0.25_DPU. However, the CPU showed the gel when more than 1 wt% content of GNP was added. The gel was observed even at low contents of GNP in the DPU because the crosslinking was formed at the DA linkage generated during the polyurethane forming cure process. The observed gel in the CPU was attributable to the crosslinking of PU with the functional group on the GNP surface. As mentioned earlier, the value of the $I_{D}/I_{G}$ for the GNP was found to be of a similar value with the reduced graphene oxide and the oxygen content on the GNP was 3.3 wt%, as shown in Table 2. Oxygen on the GNP exists in the forms of carboxylic acid, hydroxyl, and epoxide and has been demonstrated by X-ray photoelectron spectroscopy measurements in several papers [28,29], which can react with isocyanate groups of PU prepolymers and form gels at high loadings of GNPs.

### 3.3. Thermally Self-Healing Properties of GNP/PU Nanocomposites

**Figure 8:**
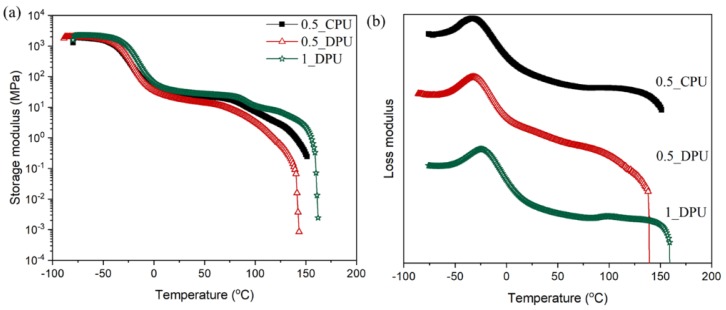
Results of DMA measurements of the CPU and DPU with 0.5 wt% and 1 wt% of GNP: (**a**) Storage moduli; (**b**) Loss moduli.

**Figure 9:**
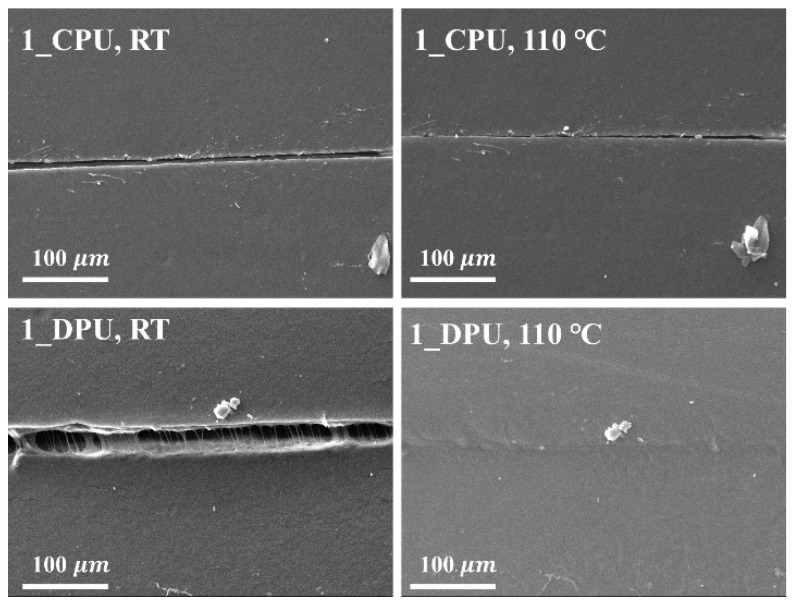
SEM images of 1_CPU and 1_DPU before (**left**, RT) and after the heat treatment (**right**, 110 °C) for 1 h.

**Figure 10:**
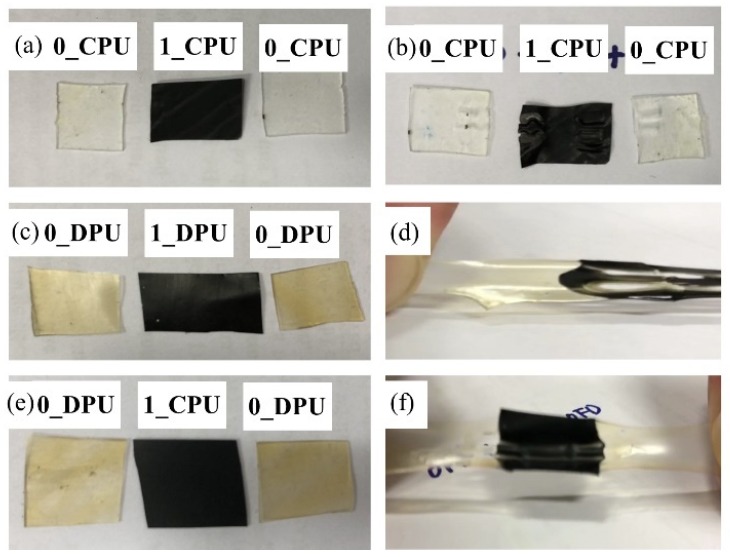
Digital images of the adhesion tests for the CPU and DPU after heat treatment at 110 °C: (**a**) Specimens of 0_CPU and 1_CPU before the test; (**b**) Specimens of 0_CPU and 1_CPU after the heat treatment and stretching; (**c**) Specimens of 0_DPU and 1_DPU before the test; (**d**) Specimens of 0_DPU and 1_DPU after the heat treatment and stretching; (**e**) Specimens of 0_DPU and 1_CPU before the test; (**f**) Specimens of 0_DPU and 1_DPU after the heat treatment and stretching.

Figure 8 presents DMA data of the nanocomposites as a function of temperature. The initial moduli in glassy states showed similar values for the three composites. As mentioned in Figure 7b, 0.5_DPU and 1_DPU are network composites with a gel content of more than 70%. Nevertheless, the DPU showed the decrease of storage modulus above rubbery plateau region like a linear CPU. Among composites with the same GNP content, the storage modulus of DPU decreased at lower temperatures. The decrease of storage modulus of 1_DPU was observed in a similar temperature range. This phenomenon is due to the retro DA reaction, which presents characteristics similar to linear PU with dissociation of physical crosslinking of hard segments at elevated temperatures. Therefore, DPU exhibited the characteristic self-healing properties, as shown in Figure 9 and Figure 10.

The specimens were scratched with a razor blade and thermally self-healing properties of the nanocomposites were investigated after the heat treatment at 110 °C on a heating plate for 1 h. Figure 9 shows the representative SEM images of the scratched specimens before and after the self-healing tests. SEM images of the scratched specimens before and after the self-healing tests for CPUs and DPUs with different GNP contents are given in Figure S3. The scratch remained in CPUs, while that of DPUs disappeared gradually within 1 h. The scratch-healing phenomenon was imparted to 1_DPU by the retro DA reactions. The reversible covalent bonds also allowed the adhesion between the films at elevated temperature via retro DA reactions. Figure 10 shows the results of the adhesion test among the films in which the films were stacked together, fixed with a clip, then treated at 110 °C for 1 h, and finally left at 60 °C for 1 h. After the samples were cooled to RT, both sides of the films were stretched to confirm the adhesion. Figure 10a,b shows the result of the adhesion test of 0_CPU and 1_CPU, in which the DA reaction could not occur. In Figure 10b, specimens were pulled apart as soon as the clip, that was used to hold the specimens, was removed. Figure 10c,d show the result of the adhesion test of 0_DPU and 1_DPU, where the DA reactions and retro DA reactions could occur. The samples of DPUs could be stretched due to the strong adhesion between specimens via the retro DA reactions and DA reactions. In Figure 10e,f, samples of 0_DPU and 1_CPU also could be stretched by the strong adhesion between the specimens. This result implies that the GNP in 1_CPU and furfuryl group in 0_DPU also underwent a DA reaction at the interfaces to implement the interfacial adhesion.

## 4. Conclusions

In this study, thermally self-healable GNP/PU nanocomposites were synthesized successfully via an in-situ DA reaction of GNP and FD as a chain extender of PU prepolymers. The interfacial tension between each material was calculated. In addition, the conditions of the DA reaction between GNP and the furfuryl group during the urethane forming cure process were obtained. DA and retro DA reactions were confirmed using the FT-IR spectrum and DSC thermogram. The mechanical strength and chemical resistance were improved because of the crosslinking of the GNP in the polymer. The scratch-healing ability was confirmed at 110 °C via the DA and retro DA reactions, and adhesion among the bulk sheets was possible. Therefore, the development of thermally self-healable nanocomposites through a bulk in-situ DA reaction is beneficial and, because of its convenience, this development could create a widening in the potential applications of polymers. 

## Figures and Tables

**Table 1:** Interfacial tensions between the constituents of graphene-nanoplate/polyurethane (GNP/PU) nanocomposites.

Materials	Interfacial Tension (dyne/cm)	
	Wu	Owen–Wendt
Prepolymer/FD	5.12	7.60
GNP/FD	3.31	4.25
GNP/Prepolymer	0.56	1.11

**Table 2:** Summary of the energy-dispersive X-ray (EDX) data for collected GNP.

Elements	C	N	O
	(wt%)	(wt%)	(wt%)
GNP	97.2	0	3.3
0.1_CPU	90.2	1.5	8.4
0.1_DPU	79.1	2.9	18.0

## Supplementary Materials

The following are available online at https://www.mdpi.com/2079-4991/9/3/434/s1, Table S1: Sample code and compositions of the GNP/PU nanocomposites, Figure S1: TGA thermogram of the GNP and modified GNP (a), Raman spectra of GNP and modified GNP(b), Table S2: Contacts angles of liquids on the surface of solid (20 °C), Table S3: Components of the surface tensions (20 °C), Figure S2: Stress–strain curves of the CPUs, Figure S3: SEM images of various GNP/PU nanocomposites before and after the healing at 110 °C for 1 h.
