# Supplementary material for: Thermally Self-Healing Graphene-Nanoplate/Polyurethane Nanocomposites via Diels–Alder Reaction through a One-Shot Process
Source: Nanomaterials (Basel). 2019 Mar 14;9(3):434. doi: 10.3390/nano9030434 (PMC6474008; doi:10.3390/nano9030434)
Supplement: Supplementary file 1 [file nanomaterials-09-00434-s001.pdf]

Supplementary Materials

Thermally self-healing graphene-nanoplate/polyurethane nanocomposites via Diels–Alder reaction through a one-shot process

Cho-Rong Oh [1,†], Sang-Hyub Lee [1,†], Jun-Hong Park [2,*] and Dai-Soo Lee [1,*]

1 Division of Semiconductor and Chemical Engineering, Chonbuk National University, Baekjedaero 567, Jeonju 54896, Korea; ohcho38@naver.com (C.-R.O.); shlee87@jbnu.ac.kr (S.-H.L.)

2 R & D Center, Lotte Advanced Materials, Sandan-ro 334-27, Yeosu 59616, Korea

* Correspondence: jh711.park@lottechem.com (J.-H.P.); daisoolee@jbnu.ac.kr (D.-S.L.); Tel.: +82-63-270-2310 (D.-S.L.); +82-61-689-1731 (J.-H.P.)

† These authors contributed equally to this work.

Table S1. Sample code and compositions of the GNP/PU nanocomposites

Sample code	Composition (wt%)				
	MDI	Polyol	BD	FD	GNP
0_CPU	31.71	63.42	4.87	0	0
0.1_CPU	31.68	63.35	4.87	0	0.10
0.25_CPU	31.63	63.25	4.87	0	0.25
0.5_CPU	31.55	63.09	4.87	0	0.49
1_CPU	31.37	62.73	4.87	0	1.04
2_CPU	31.04	62.08	4.87	0	2.01
0_DPU	29.75	59.50	0	10.75	0
0.1_DPU	29.72	59.44	0	10.75	0.09
0.25_DPU	29.67	59.33	0	10.75	0.25
0.5_DPU	29.59	59.17	0	10.75	0.50
1_DPU	29.40	58.80	0	10.74	1.06
2_DPU	29.10	58.20	0	10.74	1.98

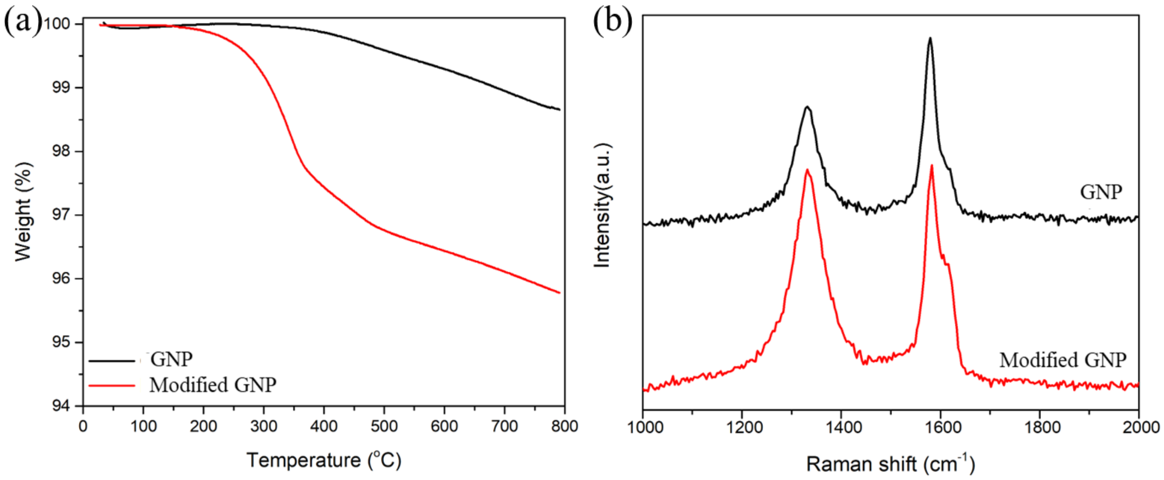

Figure S1. TGA thermogram of the GNP and modified GNP (a), Raman spectra of GNP and modified GNP (b).

Table S2. Contacts angles of liquids on the surface of solids (20 ℃)

Contact angle (°)	Deionized water	Toluene	FD
Slide glass	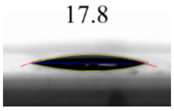	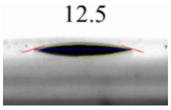	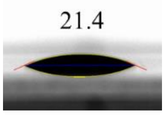
Teflon film	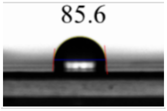	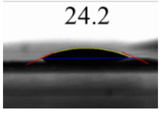	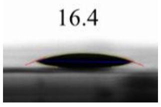
GNP	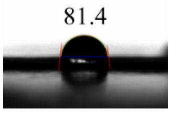	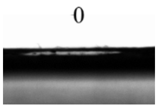	-
Prepolymer	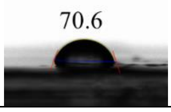	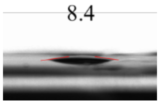	-

Components of surface tensions for Slide glass and Teflon films were determined employing equations (2) and Young's equation with contact angles of two known liquids, deionized water and toluene, to get surface tension components of FD. Components of surface tensions for GNP and Prepolymer were obtained also employing equations (2) and Young's equation with contact angles of the two known liquids. Components of surface tensions obtained are summarized in Table S3.

Table S3. Components of the surface tensions (20 ℃)

Materials	γ_i (dyne / cm)	γ_i^d (dyne / cm)	γ_i^p (dyne / cm)
Toluene[a]	28.5	27.2	1.3
Deionized water[a]	72.8	21.8	51
Slide glass	72.8	29.2	43.6
Teflon film	36.1	27.3	8.8
Prepolymer	44.8	29.6	15.2
GNP	39.9	29.9	9.9
FD	38.4	34.2	4.2

a) γ_i, γ_i^d, and γ_i^p of toluene and deionized water were obtained from literatures [1,2].

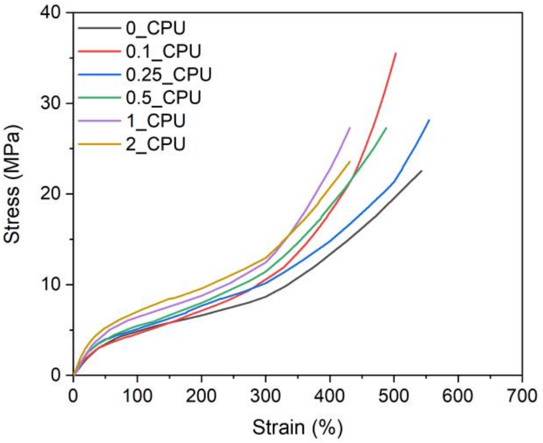

Figure S2. Stress–strain curves of the CPUs.

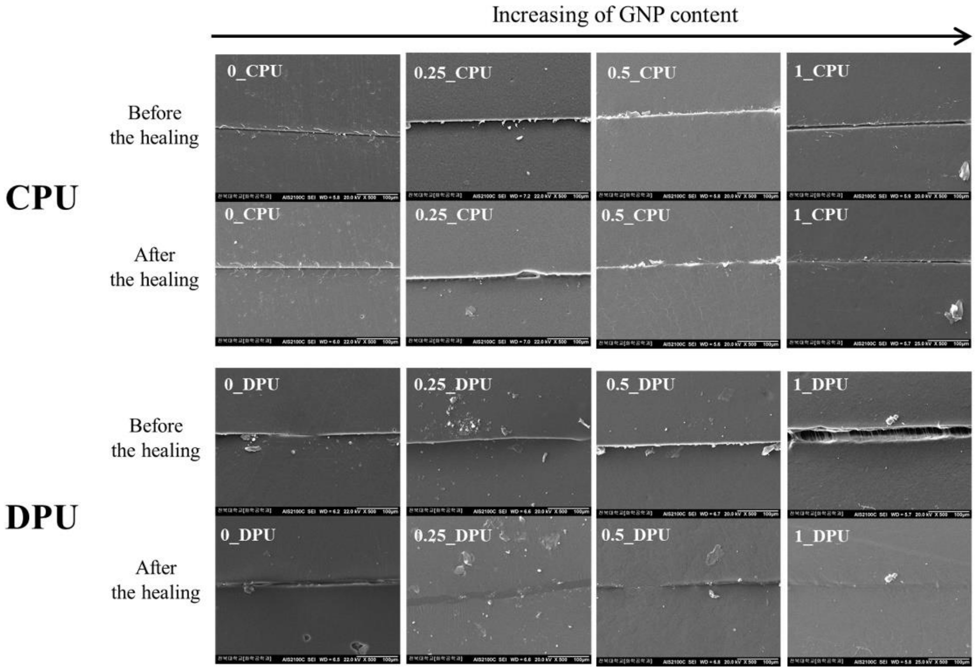

Figure S3. SEM images of various GNP/PU nanocomposites before and after the healing at 110 ℃ for 1 h.

Reference

1. Moncayo, D.; Buitrago, G.; Algecira, N. The surface properties of biopolymer-coated fruit: A review. *Ingeniería e Investigación* **2013**, *33*, 11-16.
2. Van Oss, C.J.; Ju, L.; Chaudhury, M.K., Good, R.J. Estimation of the polar parameters of the surface tension of liquids by contact angle measurements on gels. *J. Colloid Interface Sci.* **1989**, *128*, 313-319.
